# Supplementary material for: Contextual factors and spatial trends of childhood malnutrition in Zambia
Source: PLoS One. 2022 Nov 3;17(11):e0277015. doi: 10.1371/journal.pone.0277015 (PMC9632925; doi:10.1371/journal.pone.0277015)
Supplement: Attachment — Submitted filename: Authors_Rebuttal.pdf [file pone.0277015.s001.pdf]

PONE-D-21-18211
Contextual Factors and Spatial Trends of Childhood Malnutrition in Zambia

**Journal Requirements**		
**Remarks**	**Author's Response**	**Reference**
Please ensure that your manuscript meets PLOS ONE's style requirements, including those for file naming. The PLOS ONE style templates can be found at https://journals.plos.org/plosone/s/file?id=wjVg/PLOSOne_formatting_sample_main_body.pdf and https://journals.plos.org/plosone/s/file?id=ba62/PLOSOne_formatting_sample_title_authors_affiliations.pdf	Agreed.<br><br>The manuscript has been reviewed and updated to meet PLOS ONE's editorial requirements.	
We note that Figure 2 in your submission contain map images which may be copyrighted. All PLOS content is published under the Creative Commons Attribution License (CC BY 4.0), which means that the manuscript, images, and Supporting Information files will be freely available online, and any third party is permitted to access, download, copy, distribute, and use these materials in any way, even commercially, with proper attribution. For these reasons, we cannot publish previously copyrighted maps or satellite images created using proprietary data, such as Google software (Google Maps, Street View, and Earth). For more information, see our copyright guidelines: http://journals.plos.org/plosone/s/licenses-and-copyright.	Agreed.<br><br>Figure 2 was generated by the authors using publicy available data. More description has been added on the legend for all the figures.	Please see lines 338 – 343.
Please include your tables as part of your main manuscript and remove the individual files. Please note that supplementary tables (should remain/ be uploaded) as separate "supporting information" files	Agreed.<br><br>All the relevant tables have now been included in the main manuscript.	Please see lines 91, 154– 159, 213, 234
Please provide additional details regarding participant consent. In the ethics statement in the Methods and online submission information, please ensure that you have specified what type you obtained (for instance, written or verbal, and if verbal, how it was documented and witnessed). If your study included minors, state whether you obtained consent from parents or guardians. If the need for consent was waived by the ethics committee, please include this information.<br><br>Once you have amended this/these statement(s) in the Methods section of the manuscript, please add the same text to the "Ethics Statement" field of the submission form (via "Edit Submission").<br><br>For additional information about PLOS ONE ethical requirements for human subjects research, please refer to	Our study relied on secondary data from the Demographic Health Survey (DHS) repository. Upon request, they granted us the permission to access the data sets for Zambia.<br><br>Procedures and questionnaires for standard DHS surveys have been reviewed and approved by ICF Institutional Review Board (IRB). Additionally, country-specific DHS survey protocols are reviewed by the ICF IRB and typically by an IRB in the host country.<br><br>Please see https://dhsprogram.com/Methodology/Protecting-the-Privacy-of-DHS-Survey-Respondents.cfm	Please see lines 129 – 137.

http://journals.plos.org/plosone/s/submission-guidelines#loc-human-subjects-research.	More information has been provided in the manuscript.	
PLOS requires an ORCID iD for the corresponding author in Editorial Manager on papers submitted after December 6th, 2016. Please ensure that you have an ORCID iD and that it is validated in Editorial Manager. To do this, go to 'Update my Information' (in the upper left-hand corner of the main menu), and click on the Fetch/Validate link next to the ORCID field. This will take you to the ORCID site and allow you to create a new iD or authenticate a pre-existing iD in Editorial Manager. Please see the following video for instructions on linking an ORCID iD to your Editorial Manager account: https://www.youtube.com/watch?v=_xcclfuvtxQ	Agreed.<br>The corresponding author's ORCHID has been submitted.	Please see the editorial manger account.
Please include captions for your Supporting Information files at the end of your manuscript, and update any in-text citations to match accordingly. Please see our Supporting Information guidelines for more information: http://journals.plos.org/plosone/s/supporting-information.	Agreed.<br><br>This has been addressed in the manuscript.	
**REVIEWER 1**		
**Remarks**	**Author's Response**	**Reference**
The manuscript is written well but with unknown reason I did not access the tables. However, the presented information is enough to forward my concerns on the paper.	We appreciate your positive remarks. Thank you for your expertise and time.<br><br>The tables have been included in the revised manuscript.	Please see lines 91, 15 4– 159, 213, 234
As the abstract is the stand alone summary of the whole work; information like the total number of participants involved in the analysis must be included. It might be editorial problem use multivariate vs multivariable appropriately; in the paper it is used interchangeably; which is not right.	Agreed.<br><br>The abstract has been rewritten to include detailsof the study population.	Please see lines 24 – 28.
Though the introduction section address key points; it needs further modification; especially the figurative expressions of each malnutrition from the global to local context must be incorporated. If possible also add the global and the national trend from the previous reports. In addition, as mentioned in the title, "contextual factors", factors associated with each malnutrition type must be addressed in the introduction section.	Agreed.<br><br>This has been rewritten in the manuscript.	Please see lines 54 - 61
In the result section, particularly the bivariate and the multivariable analysis section; most of the measure of effects are below 1 (null value for odds ratio); this is due to the selection of the reference category; why you did that? In addition, though the measure of effect is put with its 95% CI; its interpretation is a bit confusing. Just an example let me raise this one: "In 2014, children aged 48-59 months were 0.17(95% CI: 0.12–0.23) times less likely to be overweight". First, better to	Agreed.<br><br>The bivariate analyses, were used to compare socio-economic and demographic factors with childhood malnutrition. This was to help identify if there was any significant determinants of childhood malnutrition.<br><br>The odds of overweight has been rewritten and discussed appropriately.	Please see lines 198 – 203, 207 – 211, 217 – 223 and 226 – 228.

change the reference category with the smallest percentage or you have to interpret as In 2014, children aged 48-59 months were 83% (AOR: 0.17; 95% CI: 0.12–0.23) less likely to be overweight".		
In the discussion; section from lines 240-243, is it the justification given for the similarities/discrepancies of your findings and the previous findings or recommendation? I think this is recommendation and take it this to the appropriate place and put the justification for the similarities/discrepancies of the findings.	The first paragraph in our discussion summarises key findings in the context of previous studies and policy reports such as the Rural Agricultural Livelihoods Surveys. This information has further been emphasized in the manuscript.	Please see lines 246 – 249.
**REVIEWER 2**		
Overall – a well written manuscript reporting on an analysis from the DHS that looks at geographical differences in child malnutrition and sociodemographic predictors within Zambia; however, the manuscript could be strengthened by increasing the focus on these geographic variations and highlighting the importance of the sociodemographic characteristics of provinces within Zambia in relation with the outcomes.	We thank you for your review and appreciate your feedback.<br><br>More description on the provincial socio-demographic predictors have been added in the discusion section.	Please see lines 294 - 300
Abstract<br><br>The justification for the study is that there are within country variations that are important beyond the national estimates and the methods describe assessment of within province variations, however the results only provide changes in national estimates from 2013 to 2018. The results need to be updated to support the rest of the abstract, including the conclusions.<br>I do not think that there was a decrease in overweight based on the estimates provided. I would say, it remained about the same.	Agreed.<br><br>Focussing on the provincial variation of malnutrition is relevant for policy. We have updated the reults to further emphasis this important fact.<br><br>From our analyses the decrease (from 5.7% to 5.2%) was marginal and this might serve to highlight limited progress from key stakeholders as regards to adressing obesity in children.	Please see lines 24 – 28.
Introduction –<br>37- It would be good to clarify what is meant by malnutrition, which is normally used for both under and over nutrition. While undernutrition does account for childhood mortality and impairs development, the role of overweight in childhood is not as clear. You could also mention the long-lasting consequences for chronic diseases.	Agreed.<br><br>Changes made in the manuscript.	Please see lines 41 – 44
68 – Please clarify what is meant by "a relative shift in the contribution of socio-economic determinants, communicable and non-communicable diseases". Also provide a citation for this statement.	A relative shift points to the context specific changes that compound malnutrition in children.<br><br>This has been reworded.<br><br>.	Please see lines 74 - 77
Methods –<br>84 – there seems to be a typo "of women aged of selected households".<br>84- please clarify the concept of biological children, was this asked during a screening? Were adopted children excluded?	Agreed.<br><br>These statements have been rewritten in the manuscript.	Please see the lines 83 – 89, 129 – 136.

86- Was child weight and height measured or reported by the mothers? Since the study is in children, I do not understand what is meant by women participants. Please clarify this in the study design or sample selection. Also, please distinguish between the inclusion criteria for the DHS and the inclusion criteria for this analysis.	Our study was based on scondary data collected by the DHS. The children were between the ages of 0 – 59 months. This meant their mothers had knowledge about their anthropometric measurements.	
99- The statement about malnutrition and this study including over- and under- nutrition is unclear. Please rephrase to clarify.<br><br>99-101 Perhaps this information is better suited in the introduction and/or discussion rather than in the methods.<br>Statistical analysis – is the survey designed to be representative at the province level? If so, please state it in the study description.<br>Please add a section describing the covariates for the models. The case for studying these associations should be made in the introduction	Agreed.<br><br>This has been implemented in the manuscript.	Please see lines 99 - 103
Results<br>123-125 – it sounds like the variables were included in the model based on their known importance as predictors. Perhaps that is a simpler way to state this (and in general a better one than basing it on significance). (There is a typo – “interested variables” should be variables of interest).	Agreed.<br><br>Variable selection is a key ingredient for optimal statistical analysis. We elected to understand better the relationship between malnutrition outcomes and selected factors.<br><br>The typo has been corrected in the manuscript.	Please see lines 104 - 106
169 – what is meant by uncertainty? This is not defined before in the methods.	It is an ideal practice to report estimates alongside their degree of unceratinty.	Please see lines 125 - 128
The analysis of malnutrition by province needs to be expanded to describe highest and lowest prevalence of under- and over- nutrition, as well as greatest changes over time.	Agreed.<br><br>This has been implemented in the manuscript by the mapping of the spatial trends.	Please see Figure 1 and 2.
Additional models correlating geographic or province sociodemographic characteristics to malnutrition outcomes would greatly increase the value of this paper. Please consider including these additional analysis which can be performed either by multilevel modeling with a province level or simple spatial modeling using the ARCGis	Agreed.<br><br>We mapped the prevalence per province to paint a picture of any significant difference of malnutrition in 2013 and 2018.<br><br>This helps to track progress towards the attainement of SDG 2.2.<br><br>Provincial covariates would mask the trends at the district level, where policy is much needed.	Please see Figure 1 and 2.
Conclusion<br>Even though the introduction emphasizes the relevance of geographic differences, the conclusion just presents these as an afterthought and concentrates on the associations between sociodemographic characteristics and malnutrition. There is a missed opportunity to integrate these two in a discussion about the importance of geographical variation and changes over time, and what the role of the characteristics of the different provinces means. I recommend re-writing the results and discussion, and potentially	We appreciate the feedback.<br><br>This has been addressed in the manuscript and will further be addressed in another paper that advanced a geostatistical spatial-temporal model to track the changes in malnutrition.<br><br>We have also revised our manuscript throughout to lay more emphasis on the geographical differences.	Please see lines 255 – 259, 280 – 286, 320 - 322

adding more analysis to fully address the geographic differences, rather than associations that are in general expected for child malnutrition and do not inform policies or interventions.		
