# Supplementary material for: Contextual factors and spatial trends of childhood malnutrition in Zambia
Source: PLoS One. 2022 Nov 3;17(11):e0277015. doi: 10.1371/journal.pone.0277015 (PMC9632925; doi:10.1371/journal.pone.0277015)
Supplement: Attachment — Submitted filename: Rebuttal_v3.pdf [file pone.0277015.s002.pdf]

PONE-D-21-18211
Contextual Factors and Spatial Trends of Childhood Malnutrition in Zambia

**Journal Requirements**		
**Remarks**	**Author's Response**	**Reference**
The manuscript attempts to write about malnutrition in Zambia using DHA data from two different surveys.<br>Mi biggest comment is that the manuscript has an flaw that needs to be addressed: there is not a clear research question, or questions, or hypotheses.<br>The main outcome is malnutrition, but relative to what? Is it changes? Or is it identifying factors? Or identifying factors that changed over time? ?	In this paper we intended to look at the individual socioeconomic, demographic and contextual risk factors associated with malnutrition between two DHS survey periods.<br><br>As defined by the WHO, childhood malnutrition, in all its forms, includes undernutrition (wasting, stunting, underweight), inadequate vitamins or minerals, overweight, obesity, and resulting diet-related noncommunicable diseases.<br><br>More emphasis has been added in the manuscript.	Please see lines 43 -45 and 97 - 104
Based on the hypothesis/research questions, the authors have an amazing opportunity to use the scientific method to respond to the main questions they are asking. Right now, it reads as a report of the two DHA surveys, which I don't think it is suitable for publication.	Our study sought to highlights the geographical disparity of childhood malnutrition using the 2013 and 2018 ZDHS data.<br><br>Our hypothesis is that childhood malnutrition patterns change in time and space.	
ABSTRACT:<br>The abstract has the following conclusion: "The study points to key sub-populations at greater risk and provinces where malnutrition was prevalent in Zambia". The results included in the abstract explain reductions in the odds ratios of stunting, wasting, underweight and overweight. The conclusion is not related to the results included in the abstract. I suggest the authors modify the abstract in such way that the most important results from the manuscript are listed, and the conclusion is based on the most important findings of the study.	Agreed.<br><br>Our study highlights the overall trends across all the provinces and the heterogeneity between the two periods.<br><br>We have reworded our abstract to enhance its clarity.	
OVERALL: undernutrition and malnutrition are two different concepts. The authors should consider revising the manuscript and use the appropriate term when needed: https://www.who.int/news-room/fact-sheets/detail/malnutrition<br>In the introduction, the authors consistently are using "malnutrition" when referring to "undernutrition".<br>What are the associated issues with overweight and obesity during childhood? And double burden?<br>I suggest to work a bit more on working more on the " problem" that under and over nutrition has, especially within the context of LMIC, Africa and Zambia.	According to the World Health Organization, malnutrition refers to "deficiencies, excesses or imbalances in a person's intake of energy and/or nutrients."<br><br>This can result in either undernutrition or overweight.<br><br>The introduction has been refined and additional references included.<br><br>Line 76 -78 has also been reworded.	Please see lines 75 - 79

Introduction:<br>The Lancet global series has new references on unde- and over- rnutrition in LMIC. Consider including some more updated references on worldwide undernutrition: See here: https://www.thelancet.com/series/maternal-child-undernutrition-progress<br>Line 46 needs a reference.<br>Consider rewording 76-78		
METHODS:<br>Table 1 should be supplementary material.<br>Undernourishment,.<br>Consider changing to undernutrition	Agreed.<br><br>Table 1 has been removed.	
Line 148 is malnutrition? Or undernutrition? (is it also overnutrition?)	According to the World Health Organization, malnutrition refers to “deficiencies, excesses or imbalances in a person’s intake of energy and/or nutrients.”<br><br>This can result in either undernutrition or overweight.	
The tables are very large. Consider including the most important variales.<br>Where is table 3?	Table 3 is cleary annotated.	Please see line 215
Tables 2 are showing stratified ages, however only a p-value is shown when comparing the age+ stunting, wasting a the age used as a contibnuous variable? It is not clear what the P-values are comparing? Example: is it male vs female in stunted? Is it stunting for males and females? How did the authors analize the age variable?	The association between categorical variables were analysed the chi-square test.<br>The p-values highlights if the association was significant.	
Based on the main research question of this manuscript, then the authors should consuider using generalized linear mixed models, as there are two potential clsuters of data that should be accounted for as random effects. (Although I am not a biostatistician, so I would defer to other reviewers with more knowledge on this topic).<br>1) Years<br>2) Provinces.	We used a logistic regression model since our dependent variable (malnutrion) was binary.	
