# Supplementary material for: Contextual factors and spatial trends of childhood malnutrition in Zambia
Source: PLoS One. 2022 Nov 3;17(11):e0277015. doi: 10.1371/journal.pone.0277015 (PMC9632925; doi:10.1371/journal.pone.0277015)
Supplement: Attachment — Submitted filename: Response to reviewers_Oct.pdf [file pone.0277015.s003.pdf]

PONE-D-21-18211
Contextual Factors and Spatial Trends of Childhood Malnutrition in Zambia

**Remarks**	**Author's Response**	**Reference**
Abstract: The results still do not reflect the regional differences that are described in the background, methods, and conclusion. You need to describe at least some evidence of heterogeneity in the results.	Agreed.<br><br>We have added more information on the regional disparities .	Lines 35 - 38
To address concerns related to the use of different terminology to describe undernutrition or malnutrition, I suggest that you add a footnote explaining how you are using each term in each context and that you are consistent with the use across.	Agreed.<br><br>The WHO and Zambian standards have been used to define the anthropometric indicators of nutritional status i.e. stunting, wasting, underweight and overweight.	Lines 98 - 106
Please limit the tables to key variables that illustrate this study and the results. This will address the reviewer's comment that states that this looks more like a report than an analysis.	Agreed.	Please see Table 1 and 2.
Add this information to table legends (include comparison groups, dependent variable, independent variable, co-variates, how variables were analyzed, etc).:	Agreed.	Please see table 1 and 2
Tables 2 are showing stratified ages, however only a p-value is shown when comparing the age+ stunting, wasting a the age used as a contibnuous variable? It is not clear what the P-values are comparing? Example: is it male vs female in stunted? Is it stunting for males and females? How did the authors analize the age variable?The association between categorical variables were analysed the chi-square test. The p-values highlights if the association was significant. Based on the main research question of this manuscript, then the authors should consuider using generalized linear mixed models, as there are two potential clsuters of data that should be accounted for as random effects. (Although I am not a biostatistician, so I would defer to other reviewers with more knowledge on this topic). 1) Years 2) Provinces.We used a logistic regression model since our dependent variable (malnutrion) was binary.	This seems to be a verbatim repetion of the initial reviewer comments and our response.	Please see our previous responses.
